# Smart CEI Moncloa: An IoT-based Platform for People Flow and Environmental Monitoring on a Smart University Campus

**DOI:** 10.3390/s17122856

**Published:** 2017-12-08

**Authors:** Manuel Alvarez-Campana, Gregorio López, Enrique Vázquez, Víctor A. Villagrá, Julio Berrocal

**Affiliations:** Departamento de Ingeniería de Sistemas Telemáticos, Universidad Politécnica de Madrid, Avenida Complutense 30, 28040 Madrid, Spain; gregorio.lopez.lopez@upm.es (G.L.); enrique@dit.upm.es (E.V.); villagra@dit.upm.es (V.A.V.); berrocal@dit.upm.es (J.B.)

**Keywords:** Arduino, environmental monitoring, IoT, MQTT, people flow monitoring, Raspberry Pi, sensor networks, smart cities, visual analytics, Wi-Fi

## Abstract

Internet of Things platforms for Smart Cities are technologically complex and deploying them at large scale involves high costs and risks. Therefore, pilot schemes that allow validating proof of concepts, experimenting with different technologies and services, and fine-tuning them before migrating them to actual scenarios, are especially important in this context. The IoT platform deployed across the engineering schools of the Universidad Politécnica de Madrid in the Moncloa Campus of International Excellence represents a good example of a test bench for experimentation with Smart City services. This paper presents the main features of this platform, putting special emphasis on the technological challenges faced and on the solutions adopted, as well as on the functionality, services and potential that the platform offers.

## 1. Introduction

**Figure 1:**
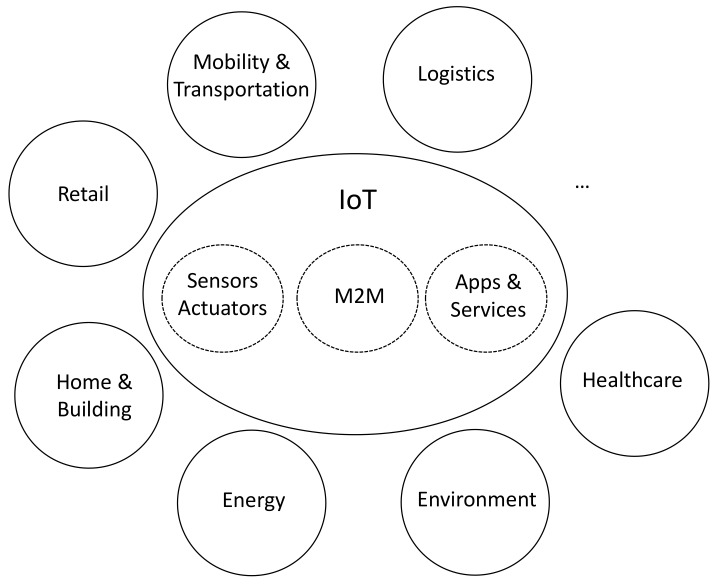
Overview of the core technologies and main application areas covered under the Smart City paradigm.

In the context of a globally accelerated urbanism process, the sustainable development of cities has become one of the major challenges of society nowadays. As a result, during the last years many initiatives and projects have been launched under the new paradigm of the so-called Smart Cities. The main goal of Smart Cities is indeed to make cities a better place to live now and in the long run, place the citizens at the center. In order to achieve this goal, Smart Cities rely on the Internet of Things (IoT) paradigm, so Information and Communication Technology (ICT) is intensively applied to all the areas related to citizens’ welfare, such as transport and mobility, healthcare, energy or the environment [1,2]. This intensive use of ICT encompasses from the massive deployment of all types of sensors and actuators and Machine-to-Machine (M2M) communications infrastructures, to the processing of the huge amount of gathered data to provide value-added services and applications, as Figure 1 illustrates.

In order to promote multidisciplinary research, development and innovation activities related to Smart Cities, in 2013 Universidad Politécnica de Madrid (UPM) launched the UPM City of the Future initiative [3]. One of the main projects within this initiative was the design, development and deployment of an IoT-based platform which allowed for the experimentation and evaluation of Smart City services in the Moncloa Campus of International Excellence (CEI Moncloa) [4].

**Figure 2:**
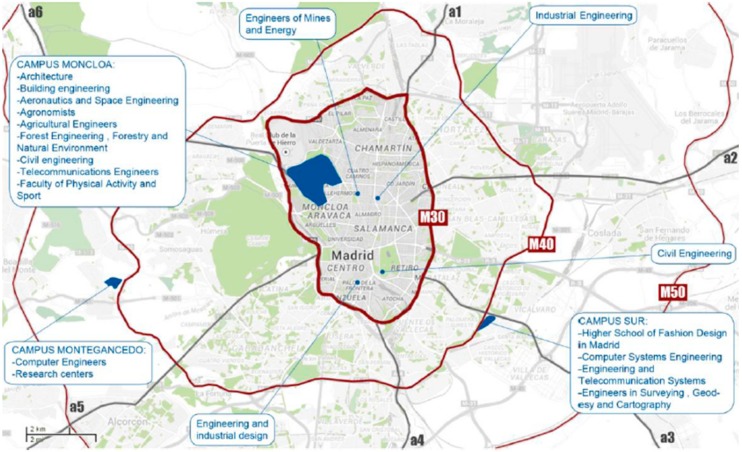
Location of Moncloa CEI Campus in Madrid.

Figure 2 shows a map of the CEI Moncloa. This campus presents some specific features that make it especially appropriate as a test bench for Smart City services. Unlike other university campuses which are in the outskirts of cities, CEI Moncloa is integrated in the metropolitan area of Madrid, the capital city of Spain. The campus covers an area of 5.5 Km^2^ and comprises 144 buildings, including schools, research centers, student residences, and sport premises. The daily flow in the campus goes up to 120,000 people and the daily road traffic accounts for thousands of vehicles, most of which do not stay in the campus but just cross it. In addition, the campus has a significant public transport infrastructure, including two underground lines and 13 bus lines.

Being an eminently university area, the industrial and commercial activity on the campus is very low. As a result, this allows performing experiments that would be very difficult to put into practice in other areas of the city without causing major inconveniences to the citizens. Thus, it is possible to set up, test and fine tune smart parking or smart lighting pilot schemes before deploying them in operational environments. Likewise, the fact that it is not a residential neighborhood facilitates the deployment of the required infrastructure during the nights or the weekends.

The main goal of this paper is to present the IoT-based platform deployed in the CEI Moncloa campus, putting special emphasis on the main technological challenges that were faced and on the solutions that were adopted, as well as on the functionality, services and potential that the platform offers.

The paper is structured as follows: Section 2 first presents an overview of the system architecture and then describes the design and implementation details of each of the subsystems which compose the platform, comparing them with related work available in the state of the art when appropriate. Section 3 goes through some of the most relevant information provided as figures or graphs by the platform in order to facilitate visual analytics. Section 4 presents three selected use cases which illustrate the potential of the platform. Section 5 discusses some key issues of IoT platforms for Smart Cities and how the IoT-based platform Smart CEI Moncloa contributes to them. Finally, Section 6 summarizes the main contributions of the paper and draws conclusions.

## 2. Architecture and Subsystem Design

As it has been mentioned in Section 1, the main goal of the IoT-based platform Smart CEI Moncloa is to facilitate the experimentation in the area of Smart Cities, allowing us to carry out studies and prove concepts which can be later put into practice in actual cities with guarantees of success. Therefore, the initial idea was to deploy a platform which incorporated the ICT infrastructure required to provide a set of basic services, along with a sensor network which allowed demonstrating the capacity and scalability of the adopted solution. Thus, the platform needed to meet the following main requirements: (1) it should be low cost and scalable; and (2) it should be based on standardized and open solutions which enable incorporating new services to the platform seamlessly.

**Figure 3:**
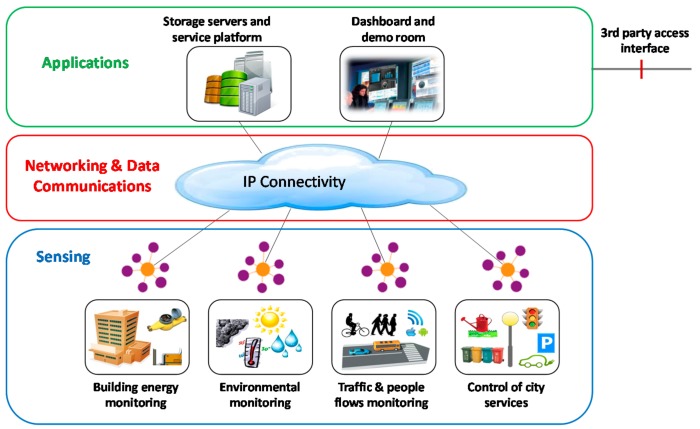
Overview of the system architecture and main layers of the Smart CEI Moncloa.

As a result, the platform Smart CEI Moncloa is based on an open architecture, aligned with the most recent standards within the scope of the Future Internet and Web engineering, which facilitates adding new functionalities and services by either UPM research groups or other companies or interested entities. Figure 3 shows an overview of the system architecture of the Smart CEI Moncloa, highlighting the sensing layer, the networking and data communications layer, and the applications or service layer, as defined by the IEEE P2413 [5,6] and other related work available in the literature [7]. This three layers are also aligned with the three domains defined in the ETSI M2M reference architecture (later transferred to OneM2M [8]), namely the device domain, the network domain, and the application domain [9].

The sensing layer is composed by a set of sensors (and eventually also actuators) that are geographically distributed across the campus. These devices are responsible for measuring the parameters of interest to the services that are delivered or the experiments that are carried out on top of the platform. Figure 3 shows some examples of sensor networks related to services that can potentially be tested and delivered in Smart CEI Moncloa, such as building energy monitoring, environmental monitoring, traffic and people flow monitoring, or control of city services (e.g., parking, lighting, watering).

The networking and data communications layer represents the core IP-based communications infrastructure which enables the secure data exchange between the sensing layer and the application layer. This layer makes the most out of the wireless and wired communications infrastructure available in the campus, as it will be described in Section 2.3.

The application layer is where the service logic and algorithms reside. It is composed of the storage servers, the service platform, and the dashboard. The storage servers are responsible for the massive storage of the data generated by the sensors and for providing access to such data to the applications that require so. They provide Big Data capabilities for handling high volumes of data, as well as Open Data format support for third party applications. The service platform is responsible for executing the applications based on SOA (Service-Oriented Architecture). The dashboard is accessible via web, which allows ubiquitous access to the control and visualization data of the platform. In addition, there is a demo room located in the Telecommunications Engineering School and equipped with big screens which allows for showing the platform capabilities to students, researchers or other interested parties. Regarding the services, Smart CEI Moncloa includes a set of initial services with the idea of progressively including new ones through its life cycle. The initial pilot services are:

- People flow monitoring, which allows counting people and associated applications, such as movement pattern analysis, places with higher transit of people, stay time in relevant places, etc.
- Environmental monitoring, which allows analyzing several environmental parameters, such as temperature, humidity, light, noise level or air composition, both indoors and outdoors.

As Figure 3 shows, each of these pilot services have an associated sensor network. Next, these sensor networks are described in detail, together with the rest of subsystems of the platform.

### 2.1. Sensing Layer

#### 2.1.1. People Flow Monitoring Sensor Network

People flow monitoring represents a topic of especial interest with a wide range of applications, which spans from crowd monitoring in events such as demonstrations or concerts, to user monitoring in public transport infrastructures such as undergrounds or airports, or client monitoring in the retail sector.

There are different ways to approach this issue. For instance, it can be performed based on video analysis [10]. Although this is a quite appropriate approach to detect crowds, it is not so appropriate for a more finely-tuned detection. In addition, this kind of solutions required the deployment of video-cameras and the processing of the recorded video on the fly. The ubiquity of mobile phones invites to consider solutions based on them. Thus, there are proposals in the state of the art that use the Global Positioning System (GPS) receiver of the phone for this purpose [11]. However, this kind of solutions present problems in indoor environments. Passive monitoring of Wi-Fi devices represents a very low cost solution for people flow monitoring that does work both indoors and outdoors [12,13,14,15,16,17,18].

This approach is based on monitoring the Wi-Fi radio frames, which include the Medium Access Control (MAC) addresses that univocally identify each device worldwide. Due to the fact that nowadays almost everybody carries a smart phone that uses Wi-Fi, it allows obtaining a reasonable estimation of the number of people in the surroundings of the listening device. It is worthwhile to mention that the Wi-Fi devices do not only transmit when they are connected to an Access Point (AP), but also when they are not, sporadically sending probe frames in order to find out if there are known APs (e.g., home) in the vicinity.

The fact that the MAC address is unique allows for establishing time correlations to compute parameters of interest, such as the average stay time of a smart phone in a given place. By using different listening devices, it is also possible to establish spatial correlations to determine, for instance, the path followed by the smartphones in a mall or underground network. Nevertheless, the Wi-Fi tracking also has drawbacks, such as that it does not allow counting or tracking people who do not use smartphones or who turn the Wi-Fi interface off. Likewise, it may also count as smart phones other devices, which may distort the measurements.

Another drawback of the Wi-Fi tracking has to do with the privacy implications of gathering MAC addresses, since they may eventually allow identifying the owners of the devices. In order to avoid privacy concerns, the companies that use this technology usually go for trade-off solutions such as announcing that Wi-Fi signals are being monitored so that people who do not want to be tracked can turn the Wi-Fi interface off. It is also common to find applications that allow requesting not to be tracked through a web form (opt-out). Some other applications go further by applying a hash function to the MAC address. Nevertheless, this anonymity technique has issues since, although the relation with the MAC address gets blurred, what happen actually is that one ID (the MAC address) is replaced by other (the hash code).

As a result, during the last years several proposals have appeared to protect Wi-Fi communications by means of MAC address anonymization. In the beginning, these proposals appeared as apps for smart phones, but during the last years the smart phone manufacturers themselves have started including these techniques in the latest versions of their OS (Operating Systems) (e.g., iOS, Android, Windows).

Such MAC address anonymization techniques aim to avoid using the actual MAC address until the device gets connected to the Wi-Fi network (i.e., they use a fake MAC address in the probe frames). The solutions depend on the manufacturer and OS [19]. Thus, in the case of iOS, the solution involves sending locally administered MAC addresses in the probe frames, randomly selecting the 3 less significant bytes of the MAC address. In the case of Android, some manufacturers have decided to use random MAC addresses in the probe frames from the MAC address ranges assigned by the IEEE to them.

In the context of Smart CEI Moncloa, privacy issues have been considered carefully. Thus, the developed Wi-Fi tracking sensors apply a hash function to the MAC addresses so that they are never stored nor processed. In addition, the data are carried securely up to the platform servers where they are handled in an aggregate manner, instead of individually.

Furthermore, the software of the developed sensors has been modified in order to avoid that the aforementioned MAC anonymization mechanisms affect the obtained measurements. Thus, the Wi-Fi frames with locally administered MAC addresses or including special MAC address ranges are discarded, so these devices are not taken into account. Anyway, it is worthwhile to mention that this is not actually such a big deal in the case of the Smart CEI Moncloa platform, since most of the users are connected to the Eduroam free Wi-Fi access, so their smart phones ends up using their actual MAC address. A similar approach is used to mitigate the effect of the MAC address anonymization techniques in malls and other public infrastructures where free Wi-Fi access is offered if service conditions are accepted.

**Figure 4:**
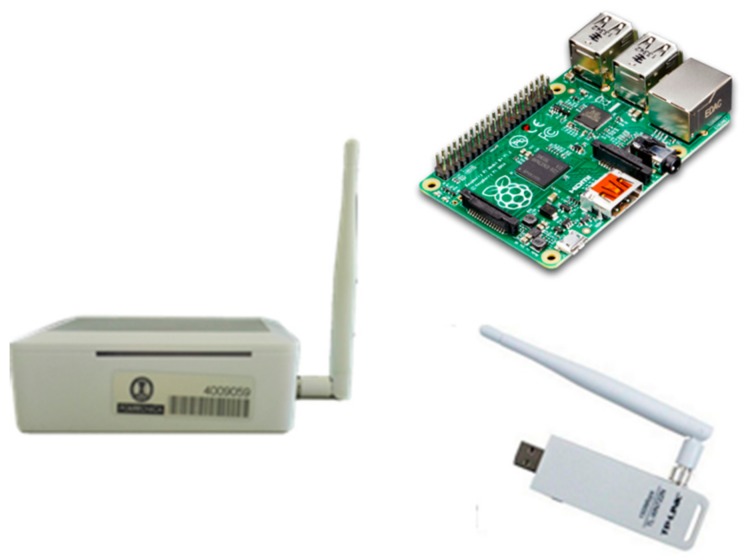
Listening device used in the people flow monitoring sensor network.

As Figure 4 shows, the Wi-Fi sensors have been developed based on a Raspberry Pi board [20] equipped with a USB Wi-Fi dongle configured in monitor mode. The cost of this solution is in the order of tens of euros, which represents a remarkable cost cut compared to other options. One of the basic software components is the wifimon program, which processes the headers of the IEEE 802.11 frames detected by the dongle on the fly. This program, inspired in the airmon-ng of the aircrack-ng suite [21], is developed in C and interacts with the Wi-Fi interface driver. By default, the program periodically scans all the Wi-Fi channels, both in the 2.4 GHz and in the 5 GHz bands, generating a report every 5 seconds including the detected devices, as well as a more detailed report on the activity of the detected devices every 15 min. Nevertheless, the channels to be monitored, the measurement time for each channel or the periodicity of the reports, can be modified through a configuration file.

It should be noted that the channel scan implies that not all the channels are monitored at a time, but they are periodically sampled, so it may happen that a frame transmitted in a channel that is not being listened at a given a moment of time is not detected. This issue is not actually relevant since the objective of the application is to detect the presence of devices during a significant period of time and in this case the probability of being detected is very high. Anyhow, the developed software allows using several Wi-Fi dongles in parallel, although this option was finally discarded for cost reasons and taking into account the good results obtained using only one Wi-Fi dongle.

**Figure 5:**
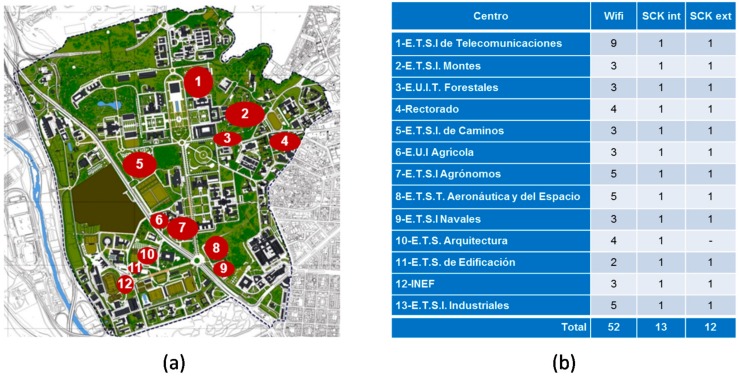
Summary of the sensors deployed in the Smart CEI Moncloa (November 2017): (**a**) Location of the schools within the Campus; (**b**) number and type of sensors per site.

The Smart CEI Moncloa platform currently comprises 52 listening devices deployed across the 13 engineering schools, as shown in Figure 5.

#### 2.1.2. Environmental Monitoring Sensor Network

If global warming and climate change were a matter of opinion at some point, it is not the case anymore. Nowadays, they represent a reality without a doubt whatsoever. As a result, environmental monitoring has become an IoT application of capital importance for sustainable development and to improve quality of life, especially in the cities [22,23,24,25,26,27].

**Figure 6:**
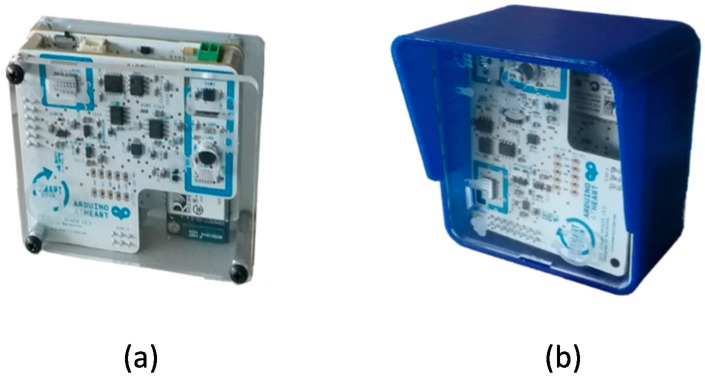
Environmental monitoring device: (**a**) Indoors; (**b**) Outdoors.

This is why it was selected as one of the initial pilot services in the Smart CEI Moncloa. The sensor network for this service is based on the Smart Citizen Kit (SCK) [28], shown in Figure 6. This device meets the main two requirements pointed out at the beginning of this section. First, it is a low cost solution (few hundreds euros) compared to expensive air quality monitoring stations (thousands of euros). Second, it is based on Arduino [29], and both the hardware and software are open source. As Figure 6 shows, a sensor shield is incorporated on top of the Arduino mother board including an array of sensors that allow measuring temperature, humidity, light, noise, CO and NO_2_. In addition, the device can be used both indoors and outdoors, if protected with the appropriate box (Figure 6b).

Being open source, the SCK allowed being finely tuned to the specific requirements of the Smart CEI Moncloa. From the software point of view, the original firmware assumes the connection to the smartcitizen.me platform [28], where the raw data from the sensors (typically electrical resistance) are processed. Such a platform fosters the involvement of citizens by allowing them to register their own devices and offers public visualization of the location of the registered nodes together with their measurements (including both real time and historical data). In the case of the Smart CEI Moncloa, the data were required to be stored in the storage servers to facilitate studies and the development of applications especially focused on the campus. Nevertheless, this was solved by developing a specific piece of software at the backend that works as gateway, since this solution offers a great trade-off between compatibility and software development effort.

In addition, the firmware of the devices was modified in order to allow automatic restarting (e.g., if the communication with the servers fails) and remote restarting upon request from the dashboard. These changes improve dramatically the operation and maintenance of the platform, since they avoid having to physically access these devices (which is typically difficult) just to hard-reset them.

**Figure 7:**
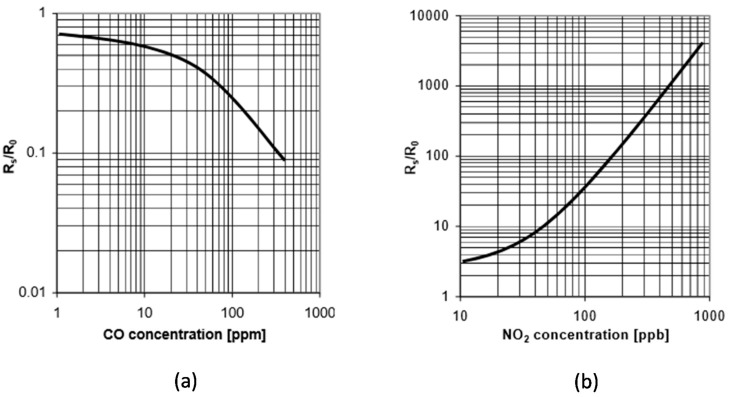
MiCS-4514 calibration curves: (**a**) CO concentration; (**b**) NO_2_ concentration [30].

The CO and NO_2_ measurements are taken by means of the MiCS-4514 MicroElectroMechanical Sensor (MEMS) [30]. This is a sensor typically used in automotive applications (e.g., to measure emissions from automobile exhausts). Hence, it is optimized for high concentration of gases, but it has attracted the interest of the IoT research community due to its very low cost. In order to obtain the concentration from the measured resistance (R_S_), the graphs shown in Figure 7 are provided. As it can be seen, these graphs represent the normalized resistance versus the concentration. However, the datasheet only provides the maximum and minimum value for R_0_ (sensing resistance in air), needed to compute the normalized resistance, whose actual value depends on specific ambient conditions (notably temperature and humidity).

**Figure 8:**
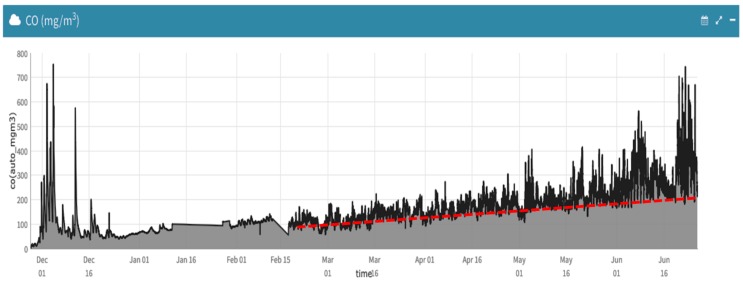
Evolution of CO concentration (mg/m^3^) from December 2015 to June 2016.

Several approaches have been explored within the context of the Smart CEI Moncloa to tackle this issue. First, the maximum and minimum R_S_ were considered as R_0_ for the CO and NO_2_ sensors respectively. However, the analyses of the gathered data revealed that the sensors somehow get contaminated with time, as Figure 8 illustrates, so it was decided to automatically update it over periods of 30 days. Although the obtained calibration is not very precise, the measurements taken by these sensors are still valuable to detect remarkable variations or trends, as it will be illustrated in Section 3 and Section 4.

As Figure 5 shows, 25 SCKs have been installed throughout the campus, 12 of them being outdoors and 13 being indoors. The main goal of the outdoor sensors is to enable the realization of environmental studies focused on the campus. Hence, they have allowed verifying significant differences in temperature and humidity across the campus, which may be caused by the Manzanares river (see Figure 2). Likewise, they have also allowed validating that there are areas with higher levels of noise and pollution, which is related to higher road traffic.

The indoor sensors have been installed in the libraries, which are one of the common places that present higher activity in the campus. In this case, the sensors are being used to monitor temperature, humidity, noise level, and air quality. In addition, this information can be combined with the information coming from the people flow monitoring service to come up with value-added applications for the university community, such as the one presented in Section 4.3.

### 2.2. Networking and Data Communications Layer

M2M communications infrastructures represent the backbone of any IoT platform, enabling the secure massive data exchange between the sensors and actuators and the information systems. M2M communications architectures can be classified into two main categories: monolithic and hierarchical. Both approaches present complementary pros and cons [31]. Hierarchical architectures are more flexible, versatile and scalable than monolithic ones, but it is also more complex to manage and secure them, since they comprise several network segments (and so several intermediate nodes) and combine different communications technologies, thus the attack surface being larger.

As it has just been mentioned, on the one side, the hierarchical architectures involve heterogeneous infrastructures which combine short range and moderate data rate technologies (e.g., WPAN technologies, such as Bluetooth or Zigbee, or WLAN technologies, such as Wi-Fi) with long range high data rate technologies (both wireless, such as cellular technologies, and wired, based on twisted pair or fiber optics). On the other side, monolithic architectures generally rely on a single technology to connect the sensors and actuators and the information systems. In this case, it is quite common that the communications infrastructure is owned by a telecom operator, which is responsible for its management and maintenance, at the expense of increasing the operational costs of the IoT platform. In this context, it is worthwhile to mention the increase popularity of LPWAN (Low Power Wide Area Networks) technologies, such as SigFox, LoRa, NB-IoT or LTE-M [32]. These technologies provide wide coverage, low consumption and low data rate, so they are especially suitable for scenarios with medium to high density of fixed sensors/actuators, as well as for applications which cover wide areas and require long-life batteries.

At application layer, apart from the option of Web Services on top of Hypertext Transfer Protocol/File Transfer Protocol (HTTP/FTP), two protocols stand out, namely: Message Queue Telemetry Transport (MQTT) and Constrained Application Protocol (CoAP) [33]. Table 1 summarizes their main features.

**Figure 9:**
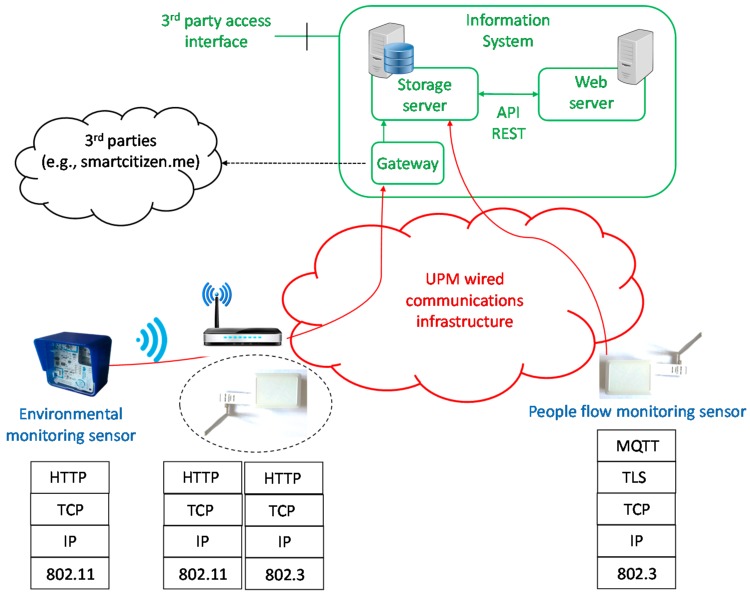
Overview of the communications architecture and protocol stack of the Smart CEI Moncloa platform.

Figure 9 shows an overview of the communications architecture and protocol stack of Smart CEI Moncloa. It can be seen that the platform presents a hybrid communications architecture, which makes the most out of the communications infrastructure available at the UPM, thus minimizing the deployment costs.

The people flow monitoring sensors (on the right hand side of Figure 9) are directly connected to the information system, located at the Telecommunications Engineering School, via the Ethernet network of the UPM. The communications are protected end-to-end by the use of Transport Layer Security (TLS) on top of Transport Control Protocol/Internet Protocol (TCP/IP). The measurements are periodically sent using MQTT. As it can be seen in Table 1, this application protocol has been selected due to the following reasons: (1) it provides reliability, since it works on top of TCP; (2) it is very lightweight; (3) the versatility and flexibility provided by the publish-subscribe mechanism itself and by the hierarchical MQTT topics. To be more precise, the publish/subscribe mechanism allows the sensors not only to send measurements periodically, but also to receive management commands (e.g., to reboot them or to perform a remote firmware update).

The implemented solution consists on the deployment of a Mosquitto MQTT broker [36] at the information system, to whom the MQTT clients installed in the people flow monitoring sensors get connected. Such clients have being developed based on the Mosquitto C library and can be easily configured using a text file. This configuration file basically sets the events which are enabled in the sensor for both publish and subscribe. In the latter case, a script can be invoked upon reception of the event (e.g., to reboot the sensor). Thus, new functionality can be easily added to the sensors.

At the information system, there are also MQTT clients that subscribe to the events of the sensors in order to gather the data to be processed. Such clients are based on the same solution explained in previous paragraph, so the aforementioned configuration files can be used to establish what to do with each event.

Taking advantage of the hierarchical structure of the MQTT topics, all the publish events follow the structure *SERVICE*/*ID*/*EVENT*(/*TIMESTAMP*). This structure allows easy storage the data coming from the sensors in a UNIX folder sorting them by service, sensor (ID), type of event and timestamp, which refers to the period when the measurement was taken. As it is explained in detail in Section 2.3, this solution has proved to be very efficient, avoiding the overhead associated to the use of databases and, at the same time, facilitating the processing of time series by using the timestamp.

In the case of the people flow monitoring sensors, the publish events start by WIFI, followed by the MAC address of its Ethernet interface, which is used as unique ID. These sensors generate different types of events. For instance, when the sensor starts working, the event *WIFI/<mac address>*/*START* is sent including a timestamp, what is useful for operation and maintenance purposes. Another example is the event *WIFI*/*<mac address>*/*SUM*, which includes a message with several counters related to the devices detected during the last 5 s, such as how many of them are active, how many have appeared, how many have gone, or how many are machines (devices that are always present). It is worthwhile to highlight also the event *WIFI*/*<mac address>*/*STA*/*TIMESTAMP* which includes a summary of the activity of the detected devices during the last 15 min. Recall that all the time intervals (e.g., 5 s, 15 min) can be configured via the configuration file.

The format of the content published under the different topics is Comma Separated Value (CSV). This is one of the most widely used data formats in IoT environments. It is especially appropriate when the structure of the sent data is fixed. In this kind of situations, it outperforms other options, such as Extensible Markup Language (XML) or JavaScript Object Notation (JSON), in terms of overhead, since CSV includes the header that explains the meaning of each field of the subsequent lines only at the beginning of the file.

As it can be observed in the left hand side of Figure 9, in the case of the environmental monitoring sensors, the communications solution was given, since they are only equipped with a Wi-Fi interface. These results in a hierarchical architecture where a given Wi-Fi network of the UPM is used to get access to the Ethernet infrastructure. It should be noted that the measurements are not encrypted end-to-end although, in the Wi-Fi link, WPA2, which used to be a very secure protocol until the publication of the vulnerabilities that have been recently found [37], together with MAC filtering is used. Additional security may be provided at application layer by means of the so-called API keys. Although in this case interoperability prevails over security, there could be scenarios where it would be of capital importance to secure this sensing infrastructure (see Section 2.1.2).

The environmental monitoring sensors assume the use of an API REST for time synchronization and for sending the measurements to the smartcitizen.me platform. Therefore, it was necessary to modify the firmware to redirect it to an API REST deployed in the information system of the Smart CEI Moncloa platform. In order to homogenize the system architecture and to make the most out of the flexibility of MQTT, such an API REST server also works as HTTP/MQTT gateway (see Figure 9). It could also be possible to install Mosquitto in the environmental monitoring sensors (e.g., [38]). However, as it was mentioned in Section 2.1.2, the adopted solution allows sharing the gathered data seamlessly with minimum changes in the sensors.

The events coming from the environmental monitoring sensors have the format *SCK*/*<mac address>*/*EVENT*. Thus, the event that is used to send the environmental parameters is *SCK*/*<mac addres>*/*RAW*/*<timestamp>*. These events include a CSV file with all the raw data measured by the sensors of the SCK. At the server, the MQTT client that processes these events store them in the subfolder *SCK*/*<mac address>*/*RAW*.

### 2.3. Application Layer

IoT applications generate unprecedented volumes of data. Therefore, data mining is key to extract valuable information from them [39], either by means of Artificial Intelligence (AI) and Machine Learning (ML) algorithms in search of patterns that allow automatically optimize processes and solve problems [40] or by means of visual representations that support data interpretation and decision making (the so-called visual analytics) [41].

As it has already been explained in Section 2.2, one of the key features of the Smart CEI Moncloa is the use of MQTT, which allows easily structuring the measurements sent by the sensors by means of folders and files at the storage server. Alternatively, such measurements could have been stored in some of the databases commonly used in IoT applications. However, as it has already been mentioned too, the measurements in the Smart CEI Moncloa represent time series and it is well-known that regular databases do not work properly when dealing with them. In order to solve this, the so-called time series databases, whose storage and search mechanisms are especially optimized for this kind of data, having being proposed in the literature [42].

Although this kind of databases could have been deployed in the storage server of Smart CEI Moncloa, for the sake of simplicity and performance, it was finally preferred taking advantage of the hierarchical structure of the MQTT topics, which include a timestamp. Such timestamps follow the YYYYMMDDhhmmss format, which dramatically facilitates the search for files by date. This feature, together with an own developed C library for handling and processing CSV files, allows developing simple scripts for time series search with very low response time. This is observed in the dashboard of the platform, where the time series queries are served very quickly, without penalizing the on-line usability of the platform, as it would happen if relational databases were in place.

In addition, the platform is processing the stored measurements in background mode and generating files with more elaborated data, which help reducing even more the query response time. Thus, in the case of the people flow monitoring sensors, for instance, daily CSV files are generated including a digest of the activity of all the devices detected throughout the day. The analysis of these files allows computing the average stay time and discriminating between machines, passers-by and stayers based on it. Another example of the benefits that this background on-the-fly processing brings, has to do with the analysis of the people flow in a whole building. In this case, the files associated to the measurements of different sensors of the same building are compared in order to avoid counting the same mobile/person several times (e.g., due to overlapping of Wi-Fi cells). The resulting file allows analyzing the people flow at building level, identifying the total stay time or the frequency of the visited places.

Finally, the dashboard of the platform is a web application developed under the Responsive Web Design (RWD) approach in order to ensure usability in all types of devices (e.g., smart phones, tablets, laptops) [43]. From the client side, the application is based on the Bootstrap framework [44], which combines Hypertext Markup Language (HTML), Cascading Style Sheets (CSS), and JavaScript. For the data representation, the well-known JavaScript libraries D3.js [45] and NVD3.js [46] are used, together with the library nvd3_graphs.js, which was specifically developed for the Smart CEI Moncloa platform. Furthermore, the JavaScript library leaflet.js [47] is used for the maps. From the server side, the application is based on Node.js [48] and Express [49]. Node.js is a well-known server platform based on JavaScript which allows developing scalable web applications (due to its event-oriented asynchronous behavior) in a fast and simple way (since clients and server are develop in the same programming language). The Express web framework makes the configuration of the Node.js server simpler.

## 3. Overview of the Provided Data

As it has just been explained, with the aim of demonstrating the functionality and potential of the deployed platform, a web-based dashboard has been developed. This application offers different functionalities depending on the user profile (namely, guess, partner, and administrator). These user profiles can visualize different graphs, maps, and tables, which are based on the data gathered by the sensor networks and facilitate performing visual analytics. In addition, the application also allows administrators to manage the deployed sensors remotely.

**Figure 10:**
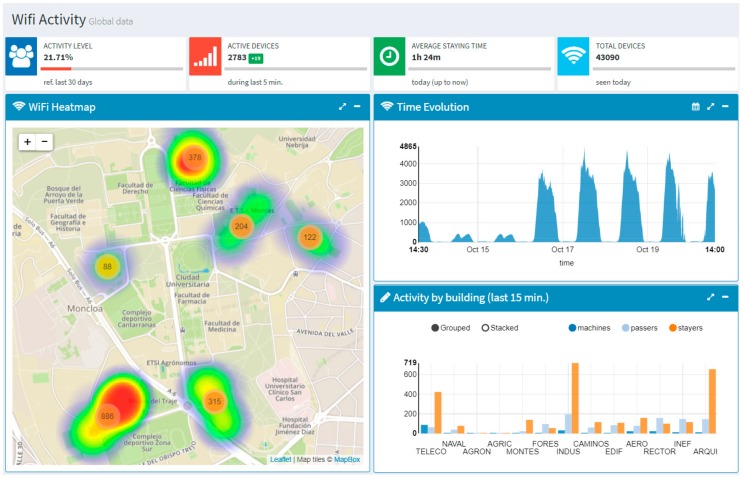
Welcome page of the Smart CEI Moncloa dashboard.

Figure 10 shows the welcome page of the dashboard [43]. This page provides an overview of the activity in the campus. On the left hand side, it can be seen a heat map that shows a real-time estimation (updated every 5 s) of the density of persons in the different premises of the campus (based on the Wi-Fi devices detected by the people flow monitoring sensor network). On the right hand side, the graph on the top shows the time evolution of the number of detected devices during the last 7 days; whereas the graph on the button shows a bar-chart that compares the number of devices detected during the last 15 min in each school. As it can be observed, the devices are classified into three categories (namely, machines, passers-by, and stayers) based on the average stay time and some heuristics. This allows differentiating between devices belonging to people who come to the school for work or studying, those that belong to people who are just passing by (e.g., couriers), and those that are actually fixed devices (e.g., printers).

**Figure 11:**
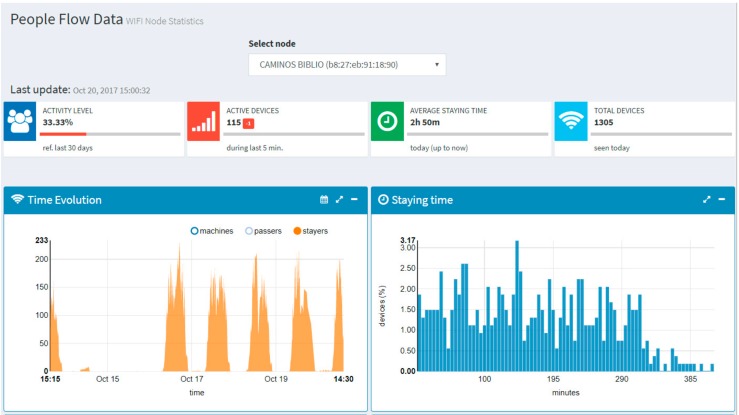
View associated to the people flow monitoring sensor installed in the library of the Civil Engineering School.

In addition, the registered users can get access to custom graphs tailored to the different schools or even to individual sensors. For instance, Figure 11 shows the view associated to the sensor installed in the library of the Civil Engineering School. In the time evolution graph (left hand side of Figure 11), the opening hours can be observed together with two occupation peaks (in the morning and in the afternoon) with an off-peak period in between (due to the lunch time). This per-sensor view also includes a histogram of the stay time (in minutes) of the Wi-Fi devices in the surroundings of the sensor (right hand side of Figure 11).

**Figure 12:**
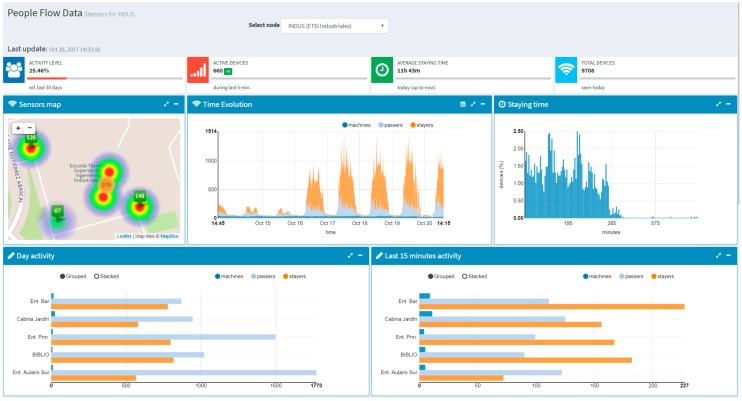
View associated to the people flow monitoring service for the Industrial Engineering School.

Figure 12 shows a snapshot of the activity in the Industrial Engineering School. In the first row of graphs, going from the left to the right, first it can be seen a heat map that illustrates the distribution of people in the different premises of the school (entrance area, classrooms, library, canteen, parking, and sport premises). Next, it can be seen a graph that shows the evolution of the number of detected devices during the last 7 days. As it has already been pointed out, these devices are classified as machines, passers-by, and stayers. The histogram of the stay time is also provided as last graph of the first row. In the second row of graphs, it can be seen two bar-charts that allow comparing the number of devices detected in each of the monitored premises. It should be kept in mind that in the building analysis the data from all the installed sensors are correlated, avoiding counting the same devices multiple times (e.g., due to Wi-Fi cell overlap).

**Figure 13:**
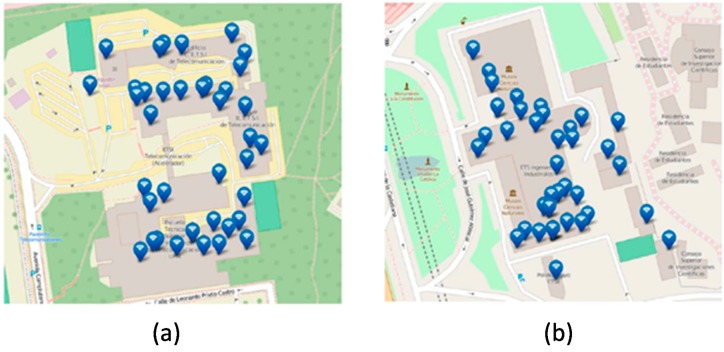

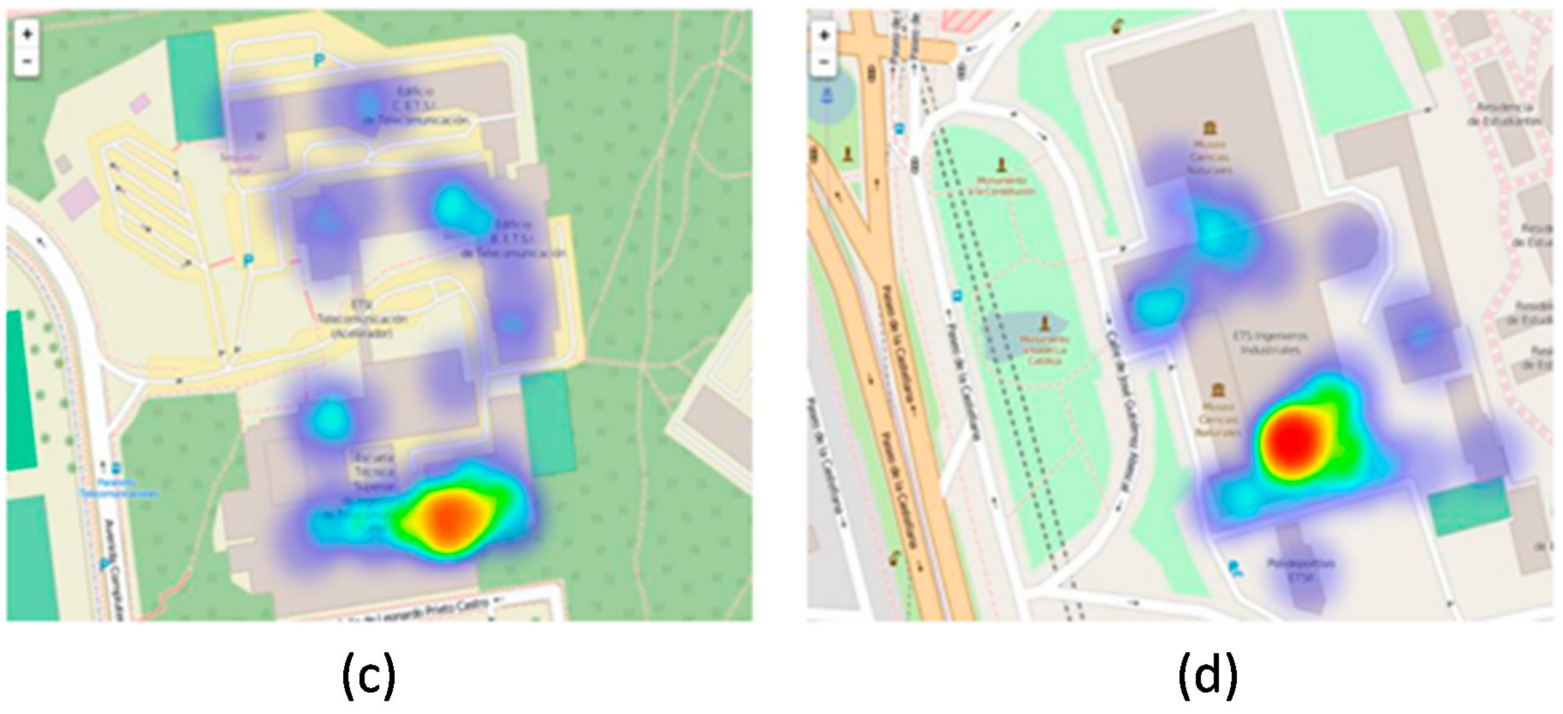
(**a**,**b**) Distribution of the people flow monitoring sensors at the Telecommunications Engineering School and at the Industrial Engineering School respectively; (**c**,**d**) Snapshot of the occupation in the different premises of the Telecommunications Engineering School and the Industrial Engineering School respectively.

Both the per-sensor and per-school views allow visualizing either real-time information or historic data. Thus, on the top of Figure 13, it can be seen the distribution of the people flow monitoring sensors at the Telecommunications Engineering School and at the Industrial Engineering School respectively; whereas on the bottom of Figure 13, it can be seen a snapshot of the occupation in the different premises of each school at a given moment of time.

**Figure 14:**
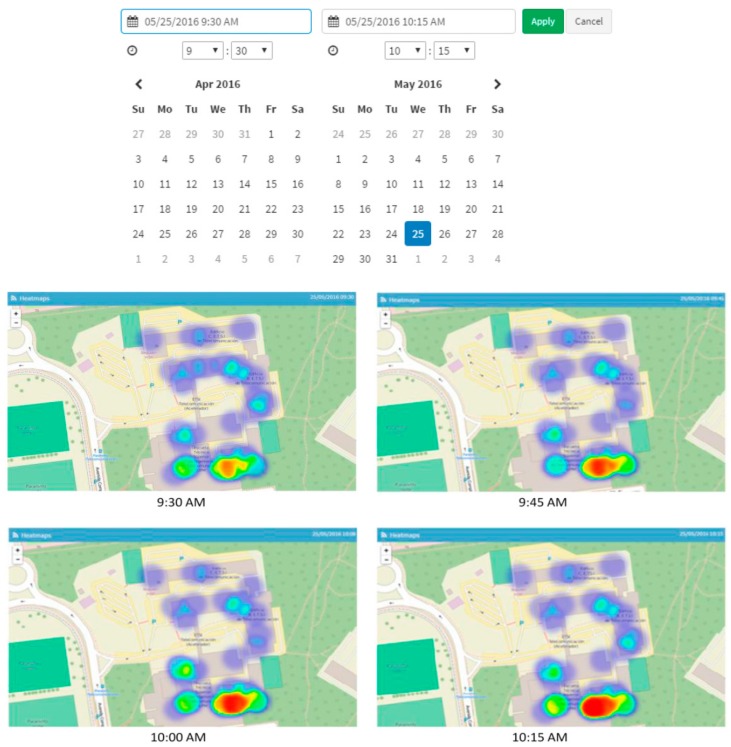
Historical occupation data for the Telecommunications Engineering School. It allows selecting the day and the time frame.

Figure 14, on the other hand, illustrates how the historical occupation data can be consulted, in this case for the Telecommunication Engineering School.

**Figure 15:**
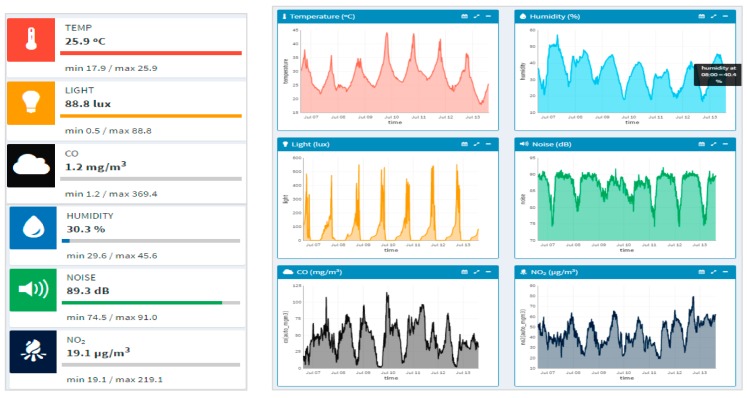
Overview of the environmental monitoring data.

To conclude this overview of the data provided by the dashboard, Figure 15 shows the view of the environmental monitoring service accessible to everybody. On the left hand side of Figure 15, it can be seen a real-time summary of the monitored parameters. On the right hand side of Figure 15, it can be seen the weekly graphs for each parameter. These graphs allow observing a clear correlation between the monitored parameters depending on whether it is day or night. It should be noted that no big difference is observed between weekdays and weekend because the libraries of the schools are open during the weekend. The registered users can also get access to the data gathered from specific sensors by selecting them.

## 4. Use Cases

The information currently provided by the Smart CEI Moncloa platform can be used and combined to come up with a wide range of value-added services for Smart Cities. This section goes through just a few examples with the aim of illustrating such a potential and fostering new ideas.

### 4.1. Use Case 1: Smart Emergency Management Application

Public safety is one of the main application areas of the people flow monitoring service. The heat maps, for instance, allow identifying the areas where there are higher concentrations of people. Thus, a potential application may focus on these areas and compare the number of detected devices with a given threshold in order to detect and avoid overcapacity. This service may also be so useful for searching for people inside buildings in case of disasters such as fire or earthquakes. This was indeed the idea that a team of UPM students presented at Hack for Good 2016 and that was awarded by Telefonica [50].

**Figure 16:**
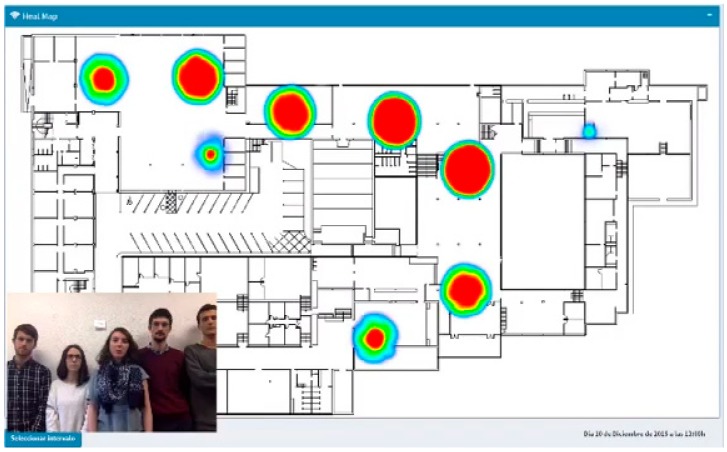
Smart Emergency Management web-based application.

The application on Smart Emergency Management was able to determine where people were located based on the data collected by the people flow monitoring sensor network of Smart CEI Moncloa. To probe the effectiveness of the solution, the students took a data set collected during December 2015 at the Telecommunication Engineering School. During the Hackaton sessions, they developed an application that processed the data in order to estimate the location of Wi-Fi devices. Figure 16 shows the developed web-based graphical user interface, which allows tracking the distribution of the devices within the building along the time.

### 4.2. Use Case 2: Traffic Restriction Application

As it has already been mentioned, environmental monitoring is of capital importance especially in the cities, where pollution is reaching levels that jeopardize citizens’ health. As a result, several important cities around the world, such as Stockholm, London, Paris or Santiago de Chile, have been applying road traffic restrictions in specific areas, such as downtown, for quite a few years. These measures are also being applied in Madrid for a few months. These measures range from forbidding the use of public parking or reducing the speed limit in the main highways surrounding the city to allowing only the entrance to the city of vehicles with odd or even license plate. The severity of the applied restrictions depends typically on the NO_2_ concentration, which is measured by a few expensive air quality monitoring stations.

**Figure 17:**
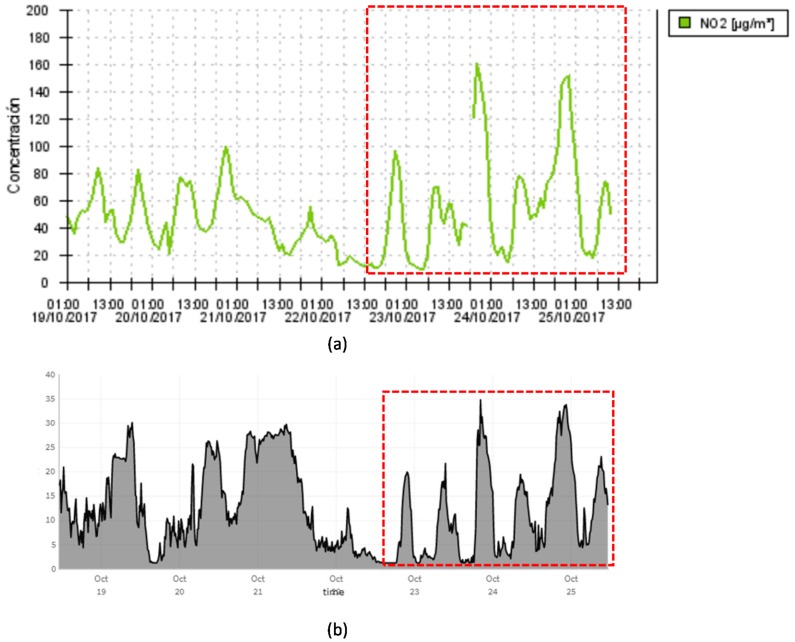
(**a**) NO_2_ concentration measured by a municipal air quality monitoring station [51]; (**b**) NO_2_ concentration measured by the outdoor environmental sensor of the Industrial Engineering School, which is in front of the municipal monitoring station.

Figure 17 compares the NO_2_ concentration measured by one of those monitoring stations with the NO_2_ concentration measured by the outdoors environmental sensor of the Industrial Engineering School, which is in front of the municipal monitoring station. It should be noted that on October 24 and October 25 the Madrid city council imposed parking and speed limit restrictions due to the pollution levels. Although, as it is explained in Section 2.1.2, the CO and NO_2_ measurements from the environmental sensors used in the Smart CEI Moncloa platform are not precisely calibrated, Figure 17 shows that they are valid for identifying sharp increases and, in general, trends.

Therefore, this kind of low-cost sensors can be combined with more expensive air quality monitoring stations to increase the granularity of the measurements, enabling more specific or focused restrictions which can be taken for less severe situations (e.g., forbid the road traffic in a specific area instead of in the whole city center and update the citizen with these restrictions dynamically via an app, which may even communicate or be embedded in the GPS navigator, thus trying to act on the traffic not only based on the traffic conditions but also on environmental variables). In this kind of hypothetical applications, security issues would need to be addressed carefully, since an attacker who got the control of a remarkable amount of low-cost sensors could provoke chaotic situations. In addition, the measurements taken by the expensive air quality monitoring stations can be used to dynamically calibrate the low-cost sensors.

### 4.3. Use Case 3: Library Application

**Figure 18:**
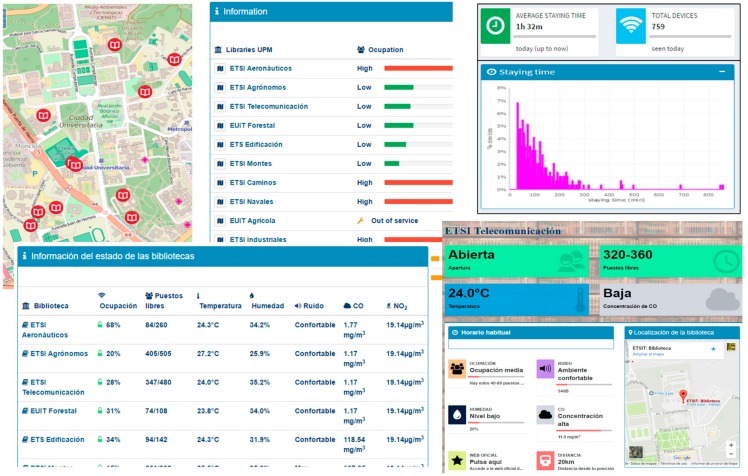
Web-based application to aid library selection.

This use case represents an example of how the information from the people flow monitoring sensors and the environmental sensors can be combined resulting in value added applications, notably to the university community in this specific case. Figure 18 shows the library application developed by a UPM student [52]. This application allows selecting one of the libraries of the different schools and provides very useful information, such as the percentage of occupation and free seats (which is an estimation based on the Wi-Fi detected devices) and the temperature, levels of noise, and CO and NO_2_ concentration (which are taken from the indoors environmental sensors). The latter information is definitely interesting to student due to the effect that these parameters have on the concentration and so on the effectiveness of the study time. This information can be combined with the distance between the users’ location and the libraries to enable trade-off selections.

## 5. Discussion

IoT-based platforms for Smart Cities involve a huge amount of sensors and actuators and a wide variety of technological features and decisions, ranging from the hardware and firmware of such sensors and actuators to the plethora of backend technologies and potential services, and the myriad of communications technologies and protocols available to connect both ends. As a result, this kind of platforms are technologically complex and putting them into practice at large scale implies huge costs and risks. Therefore, pilot schemes that allow validating proof of concepts, experimenting with different technologies and services, and fine-tuning them before deploying them massively, are especially important in this context.

The Smart CEI Moncloa presents a set of features that make it a great test bench for Smart City services. It covers a fairly large area (5.5 Km^2^) integrated into the metropolitan area of Madrid, which ensures a high traffic of people and vehicles. It involves a remarkable number of sensors (77) spread across the different schools that comprise the UPM. These sensors rely on two of the most widely used hardware platforms for IoT, notably Raspberry Pi and Arduino. The platform also uses especially relevant protocols for IoT such as MQTT and provides a human-friendly web interface based on RWD that allows consulting the available information from any kind of device (e.g., smartphone, tablet, laptop). Furthermore, the two initial pilot services (namely, people flow monitoring and environmental monitoring) are also especially relevant for Smart City scenarios, as it has been illustrated throughout the paper, and new services can be easily included.

In addition to all this, the fact that the platform is deployed in a university campus makes that the provided value goes beyond the services and applications themselves, fostering the learning and training of students, and the research and innovation from the whole university community. As a matter of fact, Section 4 presents several examples of value-added applications developed by UPM students in different contexts (e.g., hackathons, final thesis, etc.). Moreover, the Moncloa CEI includes all kind of engineering schools along with humanities schools, which make it a remarkable multidisciplinary environment, something that is so important for Smart City applications.

The lessons learned from the design, development, and deployment of the Smart CEI Moncloa can be of special interest for undertaking similar projects in the future. At the sensing layer, the paper illustrates one of the main challenges when dealing with a huge amount of low-cost sensors: keeping them properly calibrated [53]. Typically, automatic procedures to try to dynamically update and fix the calibration of the sensors will be required. In Section 2.1.2, a proposal to dynamically calibrate the data based on the gathered measurements is explained. Moreover, as it is pointed out in Section 4, if this kind of sensors are deployed together with a few high-quality sensors that drop more reliable measurements, they can be dynamically calibrated based on them. Anyway, in the case of the sensors for CO and NO_2_ concentration used in the Smart CEI Moncloa, it has been proved that, despite they are not accurately calibrated, they are still valid for detecting sharp variations and trends. As it is also mentioned in Section 4, if this kind of low cost sensor infrastructures were used for providing Smart City services, security mechanisms, such as the ones described in Section 2.2, would be necessary.

At the networking and data communications layer, taking into account the volume of devices and the associated investments that this kind of systems involve, an important takeaway is that, despite there are communications technologies that are especially appropriate for Smart City applications (such as the LPWAN technologies [32]) and so must be considered, the available communications infrastructure must be used whenever possible, in order to reduce deployment costs. Thus, if a city council offers free Wi-Fi all over the city, the AP being located, for instance, in the streetlights, and they want to start offering a smart parking service, the first approach they should consider if it is possible to send the data from the parking sensors via Wi-Fi, guaranteeing the required level of reliability and without compromising the QoS (Quality of Service) of the Wi-Fi users (which seems quite improbable, since the parking sensors can be optimized so that they only transmit a small packet when a car parks or leaves). Likewise, if a city council wanted to offer an energy management service in their public buildings which are equipped with wired and wireless infrastructures, they should first consider using such infrastructure to carry the measurements and commands back and forth.

Taking into account the wide variety of devices this kind of systems involve, it is also very common that different communications technologies and protocols co-exist within the same platform. The use of gateways or middleware is key to solve this kind of issues [54,55,56,57]. Middleware platforms are also valuable to provide common policies to access the available data, thus solving security and privacy issues [58,59]. Furthermore, it is also worthwhile to remark upon related middleware platforms focused on enabling ad hoc proximity-based Device-to-Device (D2D) communications to support so-called Mobile Social Networks in Proximity (MSNP) applications [60].

At the application layer, there are also several data formats available whose use depends on the specific features of the target application. If there are different types of devices providing heterogeneous unstructured data, JSON may be the most appropriate solution, taking into account its great flexibility and that it can be easily integrated with NoSQL databases for on-line Big Data, such as MongoDB [61]. However, if the data are heterogeneous but they follow always a fixed structure, as in the case of the Smart CEI Moncloa platform, CSV represents a great solution, since it is the most lightweight data format and meets the data requirements. The paper also presents a simple and scalable approach to store and handle time series data.

Finally, the paper also reveals that in order to obtain the maximum value from Smart City services and applications, they need to be approached holistically, since there are so many possible synergies between them. Section 4, for instance, presents the example of how combining the information from the people flow monitoring sensor network with the information from the environmental sensor network can allow developing an added-value application to aid library selection. But there are many other examples. For instance, a smart lighting service would not be actually smart if it did not take into account information related to light or to the presence of people, if available. Likewise, a smart watering service would not be actually smart if it did not take into account information related to the humidity of the ground or even the weather forecast, if available.

## 6. Conclusions

This paper presents the Smart CEI Moncloa: an IoT-based platform deployed in the Moncloa Campus of International Excellence, located in the Madrid metropolitan area, and whose main goal is to enable experimenting with Smart City services and fine-tuning them before eventually deploying them at large scale.

The platform currently offers two pilot services, namely people flow monitoring based on Wi-Fi tracking and environmental monitoring, including the following parameters: temperature, humidity, light, noise, CO concentration, and NO_2_ concentration. The paper describes the main challenges that were found and the design and implementation decisions that were made at each of the main layers of the platform, namely the sensing layer, the networking and data communications layer, and the application layer. In addition, the paper also presents the main functionalities that the platform currently offers, mostly related to performing visual analytics through a web-based dashboard which guarantees ubiquitous access, and discusses its potential by means of a few selected use cases.

As for future work, energy monitoring is the next service that is planned to be included in the platform due to its great relevance for sustainable development and to the great synergies with the already delivered services. Thus, the people flow monitoring information and the environmental information can be considered to smartly control the university Heating, Ventilation, and Air Conditioning (HVAC) systems, e.g., switching off (or properly adjusting the working point of) the heating or air conditioning when nobody is in a room or switching on the heating a couple of hours before people start arriving at the university after a very cold night or weekend. This kind of control would enable saving energy and improving comfort levels at the same time. Furthermore, the Distributed Energy Resources (DER) deployed throughout the campus (e.g., distributed generation based on solar photovoltaics and wind turbines, or electric vehicles charging spots) may be also taken into account in order to perform consumption and generation matching at campus level.

In addition, it is also planned to encourage the university community to apply ML and AI algorithms to the available data to come up with novel applications that bring value to the university itself and foster the creation of cutting-edge technological spin-offs focused on Smart Cities.

## Figures and Tables

**Table 1:** Comparison of MQTT and CoAP.

Feature	MQTT	CoAP
Standard	ISO/IEC PRF 20922 [34]	RFC 7252 [35]
Communication paradigm	Publish-subscribe	Request-response
Number of messages	16	4
Header Size	2 bytes	4 bytes
Transport layer protocol	TCP	UDP
RESTful	No	Yes

## Abbreviations

AI	Artificial Intelligence
AP	Access Point
API	Application Programming Interface
MAC	Medium Access Control
CEI	Campus of International Excellence
CoAP	Constrained Application Protocol
CSV	Comma Separated Value
D2D	Device-to-Device
DER	Distributed Energy Resources
ETSI	European Telecommunications Standards Institute
FTP	File Transport Protocol
GPS	Global Positioning System
HTTP	Hypertext Transfer Protocol
HVAC	Heating, Ventilation, and Air Conditioning
ICT	Information and Communications Technologies
IEEE	Institute of Electrical and Electronics Engineers
IP	Internet Protocol
IoT	Internet of Things
JSON	JavaScript Object Notation
LPWAN	Low Power Wide Area Network
LTE	Long Term Evolution
M2M	Machine-to-Machine
MEMS	Microelectromechanical Sensor
ML	Machine Learning
MQTT	Message Queuing Telemetry Transport
MSNP	Mobile Social Networks in Proximity
NB-IoT	Narrowband-IoT
OS	Operating System
QoS	Quality of Service
RWD	Responsive Web Design
SCK	Smart Citizen Kit
TCP	Transport Control Protocol
TLS	Transport Layer Security
UDP	User Datagram Protocol
UPM	Universidad Politécnica de Madrid
WLAN	Wireless Local Area Network
WPAN	Wireless Personal Area Network
XML	eXtensible Markup Language
